# Comparison in Deformation Behavior, Microstructure, and Failure Mechanism of Nickel Base Alloy 625 under Two Strain Rates

**DOI:** 10.3390/ma14102652

**Published:** 2021-05-18

**Authors:** Meng Liu, Quanyi Wang, Yifan Cai, Dong Lu, Tianjian Wang, Yubing Pei, Hong Zhang, Yongjie Liu, Qingyuan Wang

**Affiliations:** 1 School of Aeronautics and Astronautics, Sichuan University, Chengdu 610065, China; patricial97@163.com (M.L.); scuwangquanyi@foxmail.com (Q.W.); 2 Failure Mechanics and Engineering Disaster Prevention and Mitigation Key Laboratory of Sichuan Province, College of Architecture and Environment, Sichuan University, Chengdu 610065, China; yifan_cai1009@outlook.com (Y.C.); liuyongjie@scu.edu.cn (Y.L.); 3 Key Laboratory of Deep Underground Science and Engineering, Sichuan University, Chengdu 610065, China; 4 Sichuan Advanced Metal Material Additive Manufacturing Engineering Technology Research Center, Chengdu Advanced Metal Materials Industry Technology Research Institute Co., Ltd., Chengdu 610300, China; ludong_1786@foxmail.com; 5 State Key Laboratory of Long-Life High Temperature Materials, DongFang Turbine Co., Ltd., Deyang 618000, China; wangtianjian@dongfang.com (T.W.); peiyubing@dongfang.com (Y.P.); 6 School of Architecture and Civil Engineering, Chengdu University, Chengdu 610106, China

**Keywords:** nickel-based superalloys, tensile behavior, strain rate sensitivity, failure mechanism

## Abstract

Tensile deformation behavior and microstructure of nickel-base superalloy Inconel 625 are investigated under different strain rates of 5 × 10^−4^ s^−1^ and 5 × 10^−5^ s^−1^. According to the experimental results, yield strength and ultimate tensile strength of the alloy increase with the increase in strain rate in room temperature. Microstructure results indicate that the size of dimples is smaller in the tensile fracture surface at low strain rate than the high strain rate, and the number of dimples is also related to the strain rates and twins appear earlier in the specimens with higher strain rates. Apart from Hollomon and Ludwik functions, a new formula considering the variation trend of strength in different deformation stages is deduced and introduced, which fit closer to the tensile curves of the 625 alloy used in the present work at both strain rates. Furthermore, the Schmid factors of tensile samples under two strain rates are calculated and discussed. In the end, typical work hardening behavior resulting from the dislocations slip behavior under different strain rates is observed, and a shearing phenomenon of slip lines cross through the *δ* precipitates due to the movement of dislocations is also be note.

## 1. Introduction

Inconel 625 superalloy is extensively used as a structural material in aeronautical, petrochemical, and marine industries because of excellent high-temperature mechanical properties and resistance to severely aggressive corrosive environments especially in applications require a moderate strength at temperatures below 1200 °C [1,2,3,4]. Generally, the alloy derives its strength from the solid-solution hardening effect by adding substitutional alloying elements chromium (Cr) and molybdenum (Mo). In addition, during the aging process, in the austenite matrix at the temperature range of 600–650 °C [5], precipitation hardening of this alloy is mostly born of the precipitation of delicate metastable phase *γ* (an ordered body-centered tetragonal structure with Ni_3_Nb stoichiometry), the equilibrium phase *δ* (an ordered orthorhombic structure), formed directly during aging at 750 °C and above or by a transformation of the metastable *γ* phase [6,7], and the intergranular precipitations Laves phase also the blocky MC (M denotes Nb, Ti), M_6_C (M denotes Si, Ni, Cr), and M_23_C_6_ (M denotes Cr) carbides [8,9,10].

Strain hardening behavior is a critical factor in evaluating the plastic deformation of materials, especially for different strain rates [11,12,13]. Zheng et al. [14] studied the effect of temperature and strain rate on tensile properties of UNS N10276 and correlated the fracture modes with the mechanical properties gained from the tensile tests. Ajit K. Roy et al. [15] presented a mechanistic understanding of relation of the plastic deformation of Alloy C-276 and its temperature and strain rate. Two strengthening mechanisms in Inconel 718 alloy based on the values of strain-hardening coefficient is identified by Sundararaman et al. [7] as: (i) when n~0.85, the fracture mode exhibits shearing of *γ* precipitate particles by dislocations; (ii) when n < 0.57, the fracture mode exhibits twinning within *γ* particles. The activation of the local slip on multiple slip system is found by ALAN Xu et al. [16], which caused strain hardening during in situ micro-tensile tests. Moreover, an increase in strain rate causes an increase in flow stress for single Ni crystals during tensile test along <100> and <110> directions. Zhang et al. [16] investigated the strain hardening behavior and deformation microstructure of single crystal superalloys CMSX-4, and calculated strain hardening rate with several empirical equations. Vani Shankar et al. [3] also calculated the values of strain-hardening coefficient and exponent of thermally aged service exposed 625 alloy using experimental functions. J. Mittra et al. [17] investigated the deformation behavior of Alloy 625 in precipitated and unaged conditions, and explained the deformation phenomena with deformation microstructure and various parameters obtained from the work-hardening analysis.

Thus far, only a few works have been published that study the strain hardening behavior, and the strain rate sensitivity of Inconel 625 alloy, an empirical fitting formula of flow stress curves specially for this material is scarely seen in the open literature. The current study aims to analysis the strain hardening behavior and failure mechanism of Inconel 625 alloy through micro-tensile tests at two different strain rates and to fit the strain hardening exponent with different empirical equations and a modified ‘hybrid’ equation. Then, the electron microscopy was used to observe the fracture surface and to define the slip properties and dislocation distribution, and to study the deformation behavior and failure mechanism under both strain rates. In the following content, the experimental procedures are described in Section 2. In Section 3, the hardening exponent is calculated using a different fit equation, and deformation microstructure characterization at different strain rates about 2.0% plastic strain is observed and presented. Furthermore, the Schmid factor is calculated, and the compressive strength and deformation mechanics are analyzed. After that, the conclusion is presented in Section 4.

## 2. Experimental Procedure

**Figure 1:**
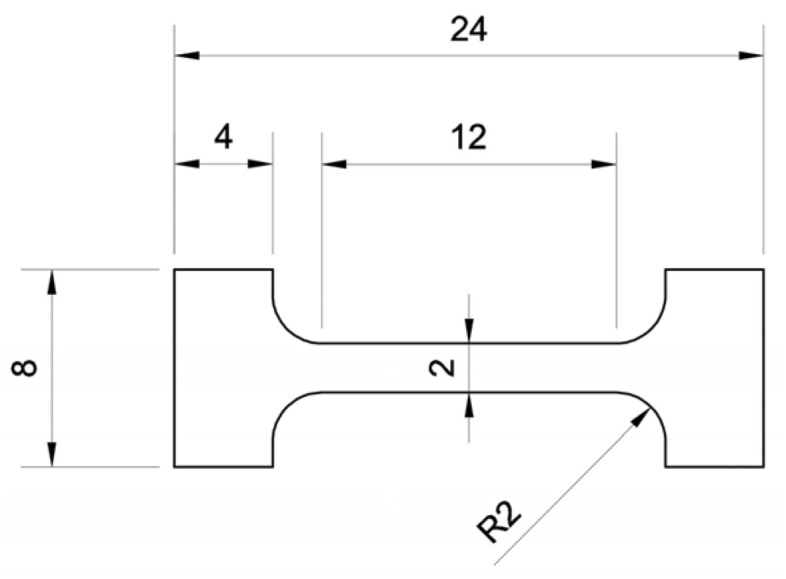
The geometry of the tensile test specimens (unit: mm).

The material was solution treated at 1200 °C for 2 h followed by water quenched immediately and the chemical composition of Inconel 625 alloy used in this work is provided by the manufacturer which is presented in Table 1. Tensile tests were performed on test system SHIMADZU AGX 100 (SHIMADZU CORPORATION, Kyoto, Japan) at two different strain rates, i.e., 5 × 10 ^−4^ s^−1^ and 5 × 10 ^−5^ s^−1^, respectively, at room temperature (RT). The micro-specimens for monotonic tensile testing were designed as the micro-plate shape referring to the international standard and previous work [16,18], as shown in Figure 1. Three specimens were tested at each strain rate, and one of the three specimens was interrupted at around 2.0% plastic strain to study the deformation structure.

To check the microstructure before the tests, specimens for microstructural observation were wet ground with 400~1200 grit SiC papers and then were etched in a solution of H_2_SO_4_ and CH_3_OH with a volume ratio of 1:4. After that, the microstructure of as-received material is detected using Quanta 450F scanning electron microscope (SEM) (FEI NanoPorts, Hillsboro, OR, USA) with HKL Channel 5 electron backscattering dif-fraction (EBSD), and FEI-TALOS F200X transmission electron microscope (TEM) (Oxford Instruments, Oxford, UK) with Super-XTM EDS detector at 200 kV voltage, respectively. In the end, morphologies of the fracture surfaces of the specimens were observed by scanning electron microscope (SEM).

## 3. Results and Discussion

### 3.1. Initial Microstructure

**Figure 2:**
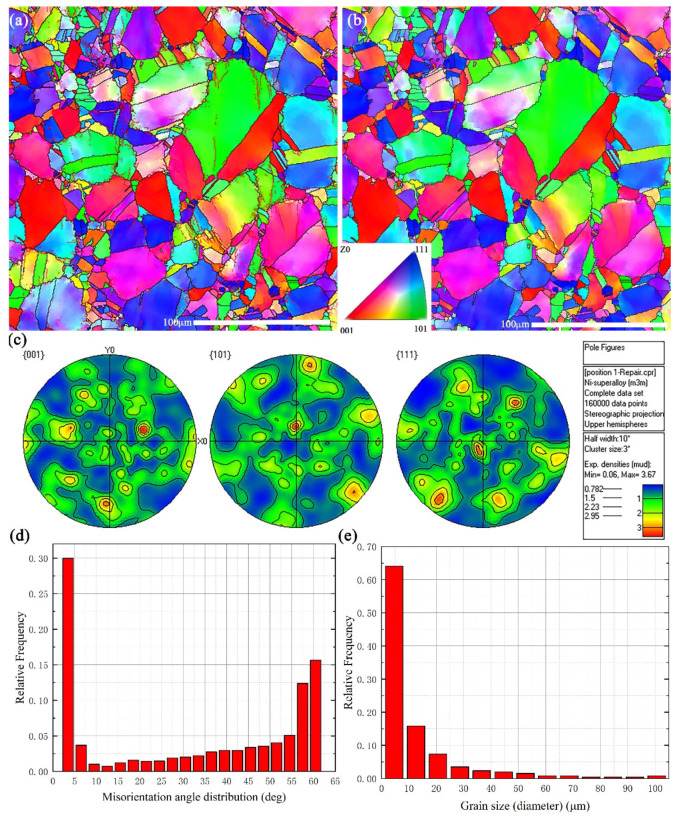
EBSD characterization of Inconel 625: (**a**) inverse pole figure with grain boundaries, (**b**) inverse pole figure with high angle grain boundaries, (**c**) pole figure, (**d**) misorientation angle distribution, (**e**) grain size distribution.

Quantification of microstructure characteristics was achieved by EBSD. Pole figures show that there is no obvious texture in the material and the statistical results show that the sample mainly consists of equiaxed grains and the average grain size is 11.61 µm. The graphic of the frequency of the grain misorientation distribution of the as-received alloy, presented in Figure 2d, exhibits a relatively high fraction of low angle grain boundaries (LAGBs, *θ* < 15°) [19] caused by the presence of substructures created by a large number of dislocations. These structures can hinder the slip of dislocations explaining to some extent the high tensile strengths of the superalloy [20]. Moreover, a significant level of misorientation about 60 deg indicates an array of twins in the as-received materials [21].

**Figure 3:**
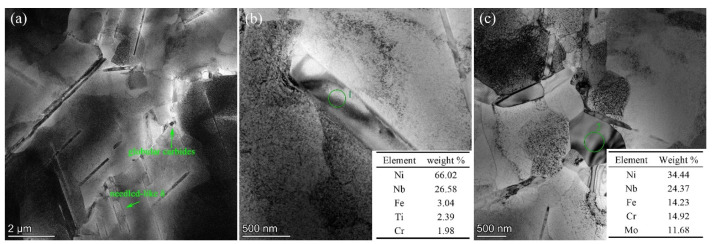
(**a**) TEM microstructure of the as-received sample; (**b**,**c**) EDS pattern of the selected areas.

Furthermore, the main composed phase in as-received Inconel 625 superalloy presented in Figure 3 by TEM images is *γ* phase, and the needled morphology distributed within the grains and at their boundaries suggests *δ* phase [22]. Apart from the needled-like *δ* phase, a globular precipitate is also be found in the intergranular regions.

To differentiate the identity of these precipitates, two areas, labeled 1 and 2, respectively, in Figure 3b,c, were selected for the TEM observations and EDS pattern microanalysis. For precipitate 1, based on its chemical analysis result, the average composition of this needled-like precipitation was determined for the major concentrations of Ni, Nb, Fe, Ti, Cr. The results confirm that it is *δ* phase [1,23,24,25]. As to the large blocky precipitate 2, the EDS analysis results of this area revealed that this polygonal shaped precipitate was Laves phase, which is consistent observed by Wang et al. [26,27,28,29]. To summarize, according to the SEM and TEM observations, it can be claimed that the *δ* phase is present in the *γ* matrix, and a few brittle phase of Inconel 625, Laves particles can be found at grains boundaries [30,31].

### 3.2. Dependence on Strain Rate of Tensile Behavior

**Figure 4:**
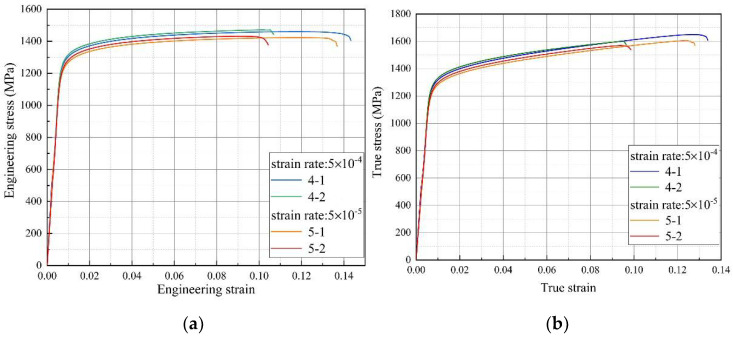
The tensile curves at different strain rates: (**a**) engineering stress–strain curves, (**b**) true stress-true strain curves.

The stress–strain curves under different strain rates were shown in Figure 4. All the specimens show a similar performance during the whole elastic stage. However, no apparent serrated flow behavior was observed on the micro-plasticity stage. As to the macro-plasticity stage, with continuous deformation, the true stress increases until fracture. It was evident that the yield strength and the ultimate tensile strength increase with increasing strain rate from 5 × 10^−4^ s^−1^ to 5 × 10^−5^ s^−1^ as illustrated in Table 2.

Besides, from Figure 4, a significant strain rate sensitivity was observed above the elastic limit. To evaluate the strain hardening behavior, the hardening capacity *H_c_* of Inconel 625 can be described as following [32],

(1) $$H_{c}\;=\;\frac{\sigma_{UTS}\;-\;\sigma_{y}}{\sigma_{Y}}\;=\;\frac{\sigma_{UTS}}{\sigma_{Y}}\;-\;1$$

Considering no obvious yield point in the stress–strain curves, *σ_Y_* is determined as the engineering stress proof 0.2% plastic deformation, and *σ_UTS_* is the ultimate tensile strength. The *H_c_* value of each specimen was calculated and listed in Table 3. However, a small gap of *H_c_* between both strain rates is not sufficient to justify the hardening capacity of the superalloy. To further confirm the strain hardening behavior of the superalloy, the strain hardening exponent (*n*) of the alloy was evaluated using several mathematical expressions, which are the most common. However, it is not surprising that these empirical equations can not accurately describe the stress–strain curves for a specified metals. Thus, the aim of this section is to study the applicability of two types (unsaturation extrapolation formula and saturated extrapolation formula) widely used fit functions [33,34] for the estimation of the *n* exponent, and to suggest an improved stress–strain fitting model.

The unsaturated model is represented by classical Hollomon [35] and Ludwik equation [13,36]. The Hollomon model is a typical full-strain model, which describes that the material strength increases in the form of power of constant hardening coefficient (*n*) during the whole process of deformation. The Ludwik model is an evolution of the Hollomon model with a fixed initial value. The extrapolated stress value of the Hollomon and Ludwik model has no upper limit, and they are written, respectively, as the following:

(2) $$\sigma \;=\;K_{1}\varepsilon^{n_{1}}$$

(3) $$\sigma \;=\;\sigma_{Y}\;+\;K_{2}{\left(\varepsilon \;-\;\varepsilon_{Y}\right)}^{n_{2}}$$

where *n*_1_ and *n*_2_ are the strain hardening exponent; *K*_1_ and *K*_2_ are the strength parameters of the superalloy; *σ* and *σ_y_* are true stress and yield stress, respectively; *ε* is the true strain and *ε_y_* used in Equation (3) is the true strain before yielding, which means the Ludwik equation only considers the true plastic deformation stage (i.e., between yield strength and ultimate tensile strength) for the curve fitting.

**Figure 5:**
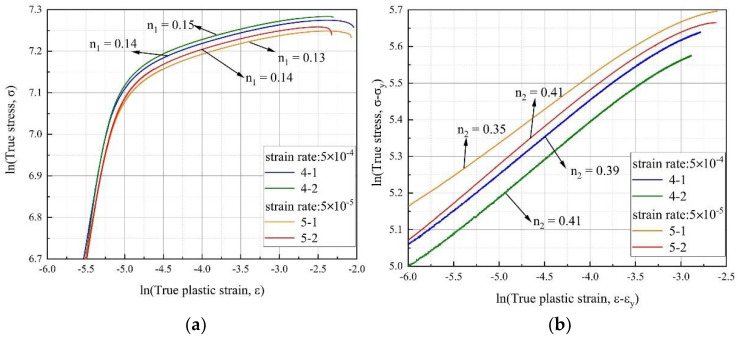
The strain hardening exponent of Inconel 625 at two strain rates: (**a**) strain hardening exponent using Equation (2), and (**b**) Equation (3).

The variations in true stress (*σ*) with the true strain (*ε*) and true plastic strain (*ε* − *ε_Y_*) were, respectively presented as double logarithmic plots in Figure 5. The hardening exponent (*n*_1_, *n*_2_) is determined as the slope of the corresponding curve, as shown in Table 3. Calculation results of *n*_1_ and *n*_2_ expose a similar rule that hardening exponent increases with increasing strain rate. This distinctly indicates that the strain hardening stages of the Inconel 625 alloy used in this work are related to the strain rate. It must be pointed out that a much higher growth of *n*_2_ at strain rate from 5 × 10^−4^ s^−1^ to 5 × 10^−5^ s^−1^, fitted by Equation (3) than that of *n*_1_ was strongly due to Equation (3) exclusive the impact of elastic deformation stage based on Hook’s law which has no contribution for strain hardening. Accordingly, the index *n*_2_ is more sensitive to the strain rate than *n*_1_ fitted by the Hollomon equation.

**Figure 6:**
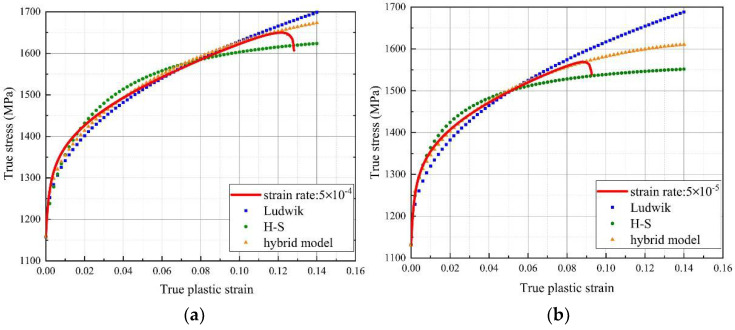
The fitting curves of Inconel 625 by different equations. (**a**) 5 × 10^−4^ s ^−1^ and (**b**) 5 × 10^−5^ s ^−1^.

Referring to the above mentioned, model with the constant initial value, i.e., start from the yield point (0, *σ_Y_*) seems to be more suitable for the hardening exponent calculation. However, as the stress increases indefinitely with the strain increases, the unsaturated extrapolation model shows deficiencies at the end of the deformation as presented in Figure 6 (blue dotted line). On this basis, saturated extrapolation model introduced the concept of hardening factor is of great significance in describing the large strain stage, in which Hockett–Sherby (H–S) [37] is a typical model, as follows:

(4) $$\sigma \;=\;\sigma_{Y}\;+\;(\sigma_{\infty }\;-\;\sigma_{Y})[1\;-\;e^{m_{1}{\left(\varepsilon -\varepsilon_{Y}\right)}^{n}}]$$

*m*_1_ is material constant; *σ*_∞_ is the stress at fracture.

The fitting curves of H–S method were shown in Figure 6. It is not hard to find that when the material starts to yield, flow stress rises rapidly, and the Ludwik equation is better fit for this stage than the H–S, then as the strain increases, unsaturated extrapolation loss accuracy, in contrast, H–S curves match better with the true case. At this point, a key problem of these above-mentioned fit equations appears: How to find a more appropriate formula, that considers both specialties of the whole variation trend. Hence, a hybrid method consists of the Ludwik and the H–S equation with a strain-dependent factor *Φ* is presented as below:

(5) $$\sigma \;=\;\sigma_{Y}\;+\;(1\;-\;\Phi )K{\left(\varepsilon \;-\;\varepsilon_{Y}\right)}^{n^{*}}\;+\;\Phi (\sigma_{\infty }\;-\;\sigma_{Y})[1\;-\;\exp(m_{2}({\left(\varepsilon \;-\;\varepsilon_{Y}\right)}^{n^{*}})]$$

where *Φ* = *cε*/*ε*_∞_, *ε*_∞_ is the strain at fracture; n^*^ is the hardening exponent; *m*_2_, *c* and *K* are the material constant. Relevant parameters of the hybrid model and the relative error are in Table 4. Error analysis was realized by taking relative error (RE) of curve integral, where the error is defined as:

$$RE\;=\;\frac{\int \left|\sigma_{FIT}\;-\;\sigma_{EXP}\right|d\varepsilon_{p}}{\int \sigma_{EXP}d\varepsilon_{p}}\;\times \;100\%$$

where, *σ_FIT_*, *σ_EXP_* are the fitted stress and experimental stress, respectively; *ε_P_* is the true plastic strain. The error results show that the curve fitted by the hybrid model has the highest approximation to the measured curve, and its relative error reaches 0.48% and 0.79% at 5 × 10 ^−4^ s^−1^ and 5 × 10 ^−5^ s^−1^, respectively.

In addition, according to the change in strain, this new hybrid model contributes to harmonizing the proportion of saturated and unsaturated equations. Obviously, the divergence of flow stress control by the Ludwik equation decreases significantly, as H–S gradually takes its effect with the increase in true plastic strain. The fit curves of these models can be seen in Figure 6, in which Ludwik and H–S fitting curves show their limitations, hybrid model, by contrast, always keeps consistent with the true curve and the hardening exponents obtained by the new method were also listed in Table 3. The largest gap of n^*^ from 0.3 to 0.35 between two strain rates indicates the material’s strain rate sensitivity, whereas a dramatic increase in stress at the latter stage of deformation leads to a higher value of *n*_2_ fitted by Ludwik equation. Analysis of exponents also confirms the applicability of this new hybrid model for specified 625 superalloys.

### 3.3. Fracture Pattern and Microstructure Morphology

**Figure 7:**
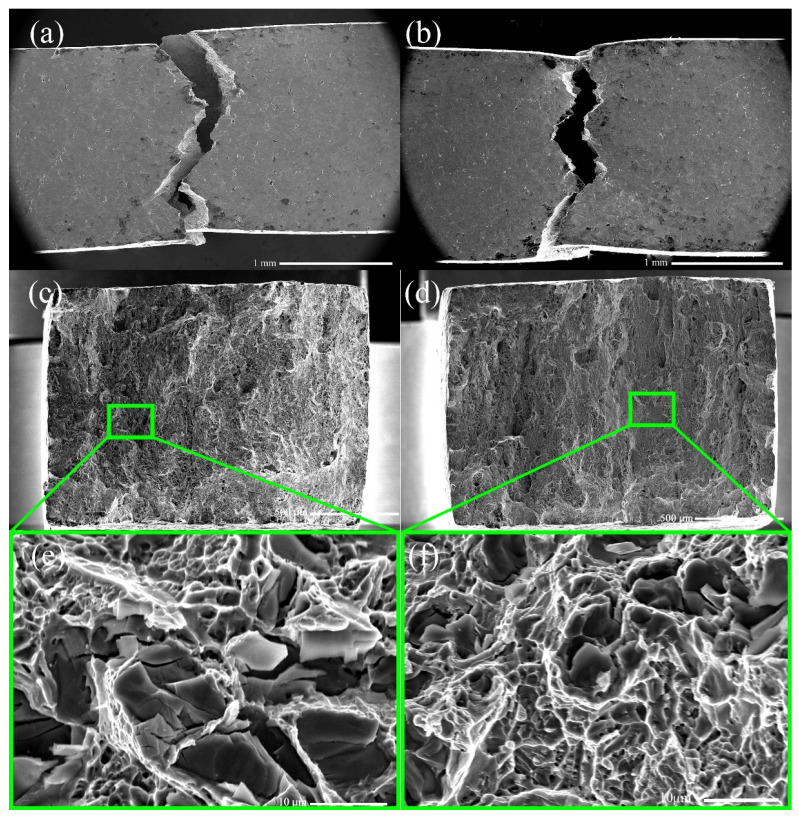
The SEM microstructure of Inconel 625 superalloys after tensile tests, fracture and magnified view of fracture (**a**,**c**,**e**) 5 × 10^−4^ s ^−1^ (**b**,**d**,**f**) 5 × 10^−5^ s ^−1^.

According to the above investigation, the tensile properties of Inconel 625 superalloy depend strongly on the strain rates, resulting in different fracture patterns at both strain rates. In that way, the representative SEM micrographs of fracture morphology of the specimens after tensile tests show quite different features, as shown in Figure 7.

A slight neck can be observed at both strain rates, as shown in Figure 7a,b, which indicates that local plasticity presents before final failure. There is a flat fracture surface with river patterns and fine and shallow dimples on the fracture surface at 5 × 10^−5^ s^−1^ presented in Figure 7d,f, which show ductile fracture mode by the occurrence of dimples on the fracture surface. Comparing to the low strain rate, a large number of transgranular cracks can be observed from the specimens destroyed under 5 × 10^−4^ s^−1^ in Figure 7c. However, the tensile fracture of high strain rate is mixed mode of fracture though the failure was predominantly intercrystalline. It should also be indicated that the dimple size increases with the increasement of the strain rate.

**Figure 8:**
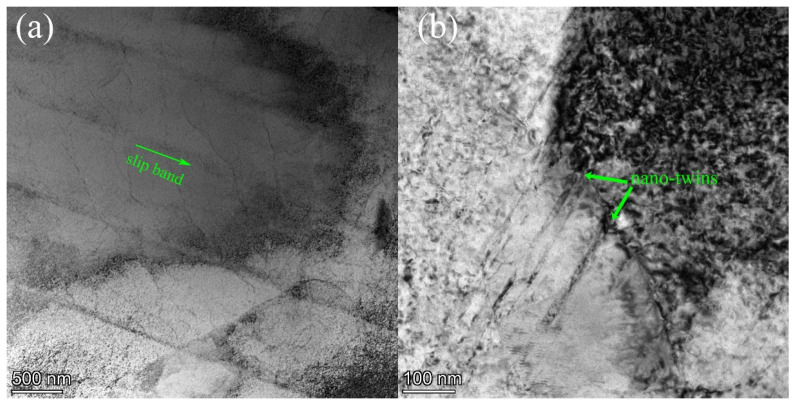
The TEM microstructure of Inconel 625 superalloys at 2.0% plastic strain. (**a**) slip band (**b**) nano deformation twin at 5 × 10^−4^ s ^−1^ sample.

To study the deformation mechanism and dislocation configuration, the TEM images of interrupted tensile tests (2.0% plastic strain excluding elastic strain) as shown in Figure 8 and are gathered for the following analysis. All the deformed samples show planar slip with high dislocation density. Some specific slip bands (Figure 8a) consisted of dislocation structure of densely packed primary and secondary dislocations was observed in the gauge length [38,39]. A few nano deformation twins are occasionally observed in several grains [40] at 5 × 10^−4^ s^−1^ without 5 × 10^−5^ s^−1^, see Figure 8b Generally, in face-centered cubic (FCC), twinning is facilitated through lower stacking fault energy (SFE) and special deformation conditions (such as low deformation temperatures or high strain rates) [41,42]. For Inconel 625, as an FCC metal with low SFE, the deformation mechanism is twinning ({111}<112>) and dislocation slip ({111}<110>) through the tensile deformation modes [43]. Therefore, slip is the main deformation mode during the initial stages of tensile deformation under both strain rates. After dislocation multiplication and tangle formation, further deformation results in dislocation cross slip being suppressed to that extent the cross slip of Shockley partial dislocations could lead to intrinsic stacking faults on parallel {111} planes, leading to twins [44]. Thus, twinning is the other deformation mode during the futher stages of tensile deformation, and twin boundaries act as strong obstacles to the dislocation motion, resulting in improvement of alloy strength [45,46]. Li et al. [47] has also found the deformation twins in other alloys, and demonstrate that this critical change of deformation mechanisms from dislocation slips to twinning behavior is responsible for such an increasing of hardening exponent (*n*) value from 5 × 10^−5^ s^−1^ to 5 × 10^−4^ s^−1^. This is consistent with our experimental results.

In addition, the typical planar slip during deformation is dicided by the Schmid factor and yield stress. With the consideration of the crystal orientation, the critical resolved shear stress (CRSS) can be determined as following with Schmid’s law [48].

(6) $$\tau_{CRSS}\;=\;\sigma_{Y}M$$

where *M* is Schmid factor.

**Figure 9:**
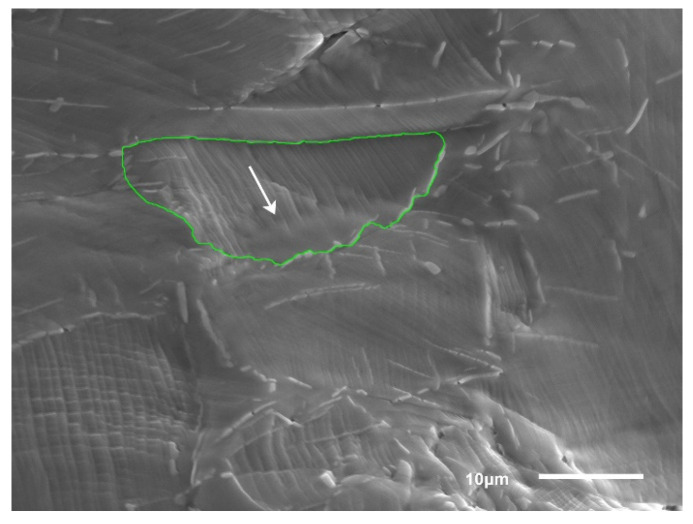
Schematic of slip direction calculated by MTEX.

To define the relationship between Schmid factor and slip, a free MATLAB toolbox MTEX [49] was used to calculate the Schmid factor and visualize the active slip systems of a given EBSD map in Figure 9. The Schmid factor on different slip systems and CRSS of a typical grain is presented in Table 5.

The slip direction calculated by MTEX (white arrow) is in accordance with the EBSD map presented in Figure 9. and the results show possible primary slip system was (−1, 1, −1) [0, −1, −1], the secondary slip system (111) [0, −1, 1] and the third slip system (−1, 1, 1) [1, 0, 1] are activated during tensile tests. Furthermore, the max CRSS value on the strain rate of 5 × 10^−4^ s^−1^ is 573.72 Mpa, which is higher than 547.91 MPa at 5 × 10^−5^ s^−1^. It indicates that CRSS value can be affected by the strain rate, which can be explained with the Taylor equation;

$$\tau \;=\;\tau_{0}\;+\;\alpha Gb\sqrt{\rho \;-\;\rho_{0}}$$

where *α* is a constant measuring the efficiency of dislocation strengthening, *G* is the shear modulus and *b* is the Burgers vector, hence it is clear that shear stress (*τ*) is directly related to the final dislocation density (*ρ*).

### 3.4. Failure Mechanism

**Figure 10:**
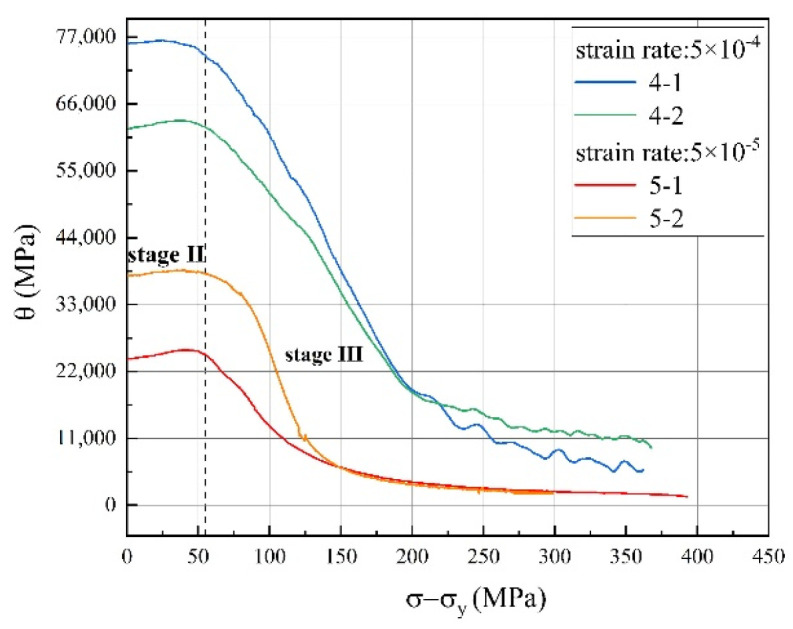
The strain hardening rate and exponent of Inconel 625 at two different strain rates.

In this section, the work-hardening concepts were induced to explain and predict the stress–strain response of the alloy from the point of dislocation theosries, and the work-hardening rate of 625 superalloy shows an increase with increasing strain rate. As seen in the Kocks–Mecking type plot of strain hardening rate *θ* (=*dσ*/*dε*); vs. net flow stress (*σ* − *σ_Y_*) at two strain rates of the Inconel 625 superalloy, as shown in Figure 10, work-hardening behaviour of this alloy is characterized by an initial sharp fall in *θ*, followed by satge II, i.e., a plateau and then a further gradual fall can be denoted as stage III work-hardening, respectively [17].

**Figure 11:**
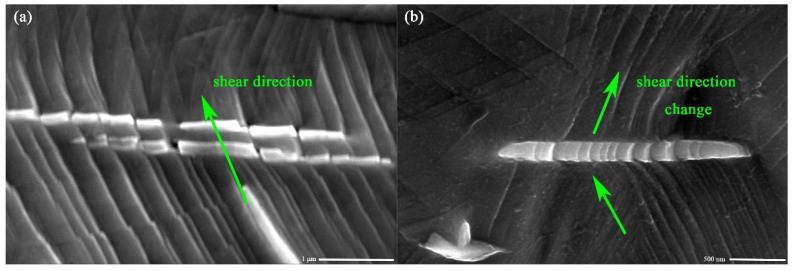
Slip line shear into *δ* phase. (**a**) 5 × 10^−4^ s ^−1^; (**b**) 5 × 10^−5^ s ^−1^.

Stage II is characterized by a high initial *θ* value that almost stabilised at a constant, and such a behaviour is attributed to an initially linear stage II strain hardening behaviour [12]. Additionally, the stress reaches the CRSS, one or more slip systems are activated in this stage. Meanwhile, dislocation shear into *δ* phase has been observed, as shown in Figure 11, which plays an important role in controlling the tensile performance of the alloy. As to stage III, the sharp drop in the slope of the sample under 5 × 10 ^−4^ s^−1^, as shown in Figure 10, occurs earlier than that of the low strain rate sample. This indicates a premature recovery process occurs on a high strain rate sample, speculated as once the recovery process starts in the specimen with higher dislocation density, it goes faster than the other [17].

Based on the above discussion, a model about strain hardening takes into account to explain the strain hardening behavior of this alloy [50],

*σ* = *σ*_0_ + *σ_HP_* + *σ_d_* (7)

where *σ*_0_ is the stress contributed by the friction; *σ_HP_* = *kd*^1/2^ is contributed by the Hall–Petch; *σ_d_* = *MαGbρ*^1/2^ is contributed by the Taylor dislocation.

During deformation, the gliding of dislocation causes plastic strain in the material. As the strain increases, the material begins to yield, dislocations nucleate, and interact, leading to dislocation density increases. Thus stress contribution caused by dislocation density can be written as the total flow stress subtracting the yield stress,

*ρ*^1/2^ ∝ *σ_d_* − *σ_y_* (8)

**Figure 12:**
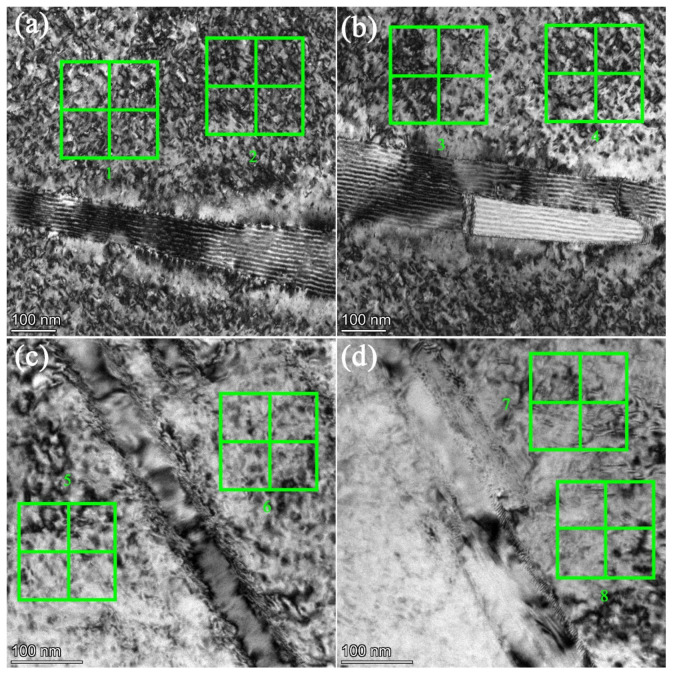
TEM micrograph used to calculate *ρ*. (**a**,**b**) 5 × 10^−4^ s ^−1^; (**c**,**d**) 5 × 10^−5^ s ^−1^.

The applied stress necessary to deformation is obviously proportional to the dislocation density in the material. Thus far, dislocation density is affirmed necessary in this investigation. The magnitude *ρ* was determined by the line intersection method [51,52,53] based on the superimposition of a grid consisting of horizontal and vertical test lines on the TEM micrographs that contained dislocations of the specimens at both strain rates. Since here we only need to compare the different influence between the two strain rates qualitatively, we can briefly distinguish the dislocation density of different strain rates by computing the average number of intersections of each test lines with dislocations. To simplify the computing process further, the grid was drawn as a square, as illustrated in Figure 12. For each strain rate, two pictures were used, and for each picture, we grid two areas that unaffected by the precipitate, then the computing results are listed in Table 6.

However, the average values of intersection number determined from the TEM micrographs of tensile specimens strained at 5 × 10^−4^ s^−1^ was relatively higher than the low stain rate ones 5 × 10^−5^ s^−1^. This can also be verified by examining the TEM micrographs presented in Figure 11, showing the denser population of dislocations at this strain rate. Essrntially, the number and velocity of dislocations are improved at a high strain rate, which accompanied with the increasing of the dislocation density per unit area. Accordingly, the high initial dislocation density in the high strain rate specimens might have contributed to the initial high and nearly constant strain hardening rate (i.e., stage II linear hardening shown in Figure 10 of the specimens under 5 × 10^−5^ s^−1^. This suggests that much higher activation energy is required for the plastic flow due to powerful barriers to the dislocation movement [54]. As described by reference [12], a positive work hardening stage II occurs due to continuous reduction in mean free path during dislocation–dislocation interaction and dislocation pileups at the grain boundary. Thus, the Taylor dislocation contribution *σ_d_* = *MαGbρ*^1/2^ in Equation (7) dominates this region. The increasing of the number of dislocations leads to the increasing of the resistance to the dislocation movement, and the stress required to deform the materials becomes higher with increasing deformation. This is in accordance with Zhang’s results in nickel-based superalloy [16].

Moreover, an interesting phenomenon worth to note is that the original shearing direction changes when dislocations slip shear through the *δ* phase under a low strain rate, see Figure 11. In contrast, under a high strain rate, shearing is always in the same direction. The following statement may interpret this strain rate related performance: When experiments were carried out at a high strain rate, with a high per unit time strain, the inside of the material is subjected to more intense deformation per unit time, which means more energy is imported to help dislocations go through the obstacle. On the opposite, low strain rate specimens cannot cross the *δ* phase directly. Therefore, shearing direction will change to the pass with the lowest energy cost [55,56,57,58].

## 4. Conclusions

Tensile tests have been conducted on nickel-base superalloy Inconel 625 under two strain rates at RT. The main conclusions derived from this study are as follows:

(1) All the strain–stress curves show a similar trend: no apparent serrated flow on the micro-plasticity stage. During the macro-plasticity stage, the true stress increases until fracture. In contrast, there are differences between different strain rates: with increasing strain rate from 5 × 10^−4^ s^−1^ to 5 × 10^−5^ s^−1^, the yield strength and the ultimate tensile strength increase.
(2) Higher strain rate also results in greater strain hardening exponents. It should also be pointed out that the equation excludes the impact of the elastic deformation stage on Hook’s law, is more appropriate to fit the strain hardening exponents *n* than the Hollomon equation. In addition, the hybrid model, integrates saturated and unsaturated methods is the most accurate for the calculation of hardening exponents.
(3) The fracture morphologies of tensile specimens depend on the strain rate. Flat fracture surface and dimples can be observed in all the deformed samples. The size of the cleavage facet and dimple increases with the increase in the strain rate.
(4) The strain hardening stages depend on the strain rate. Under a high strain rate, specimens possess a high constant work hardening value at stage II and a high slop in stage III. The original shearing direction changes when dislocations slip shear through the *δ* phase under a low strain rate, while under a high strain rate, shearing is always along the same direction.

## Figures and Tables

**Table 1:** Chemical composition (wt.%) of the Inconel 625 alloy used for the current study.

Ni	Cr	Mo	Nb	Fe	Ti	Al
residue	20.0~23.0	8.0~10.0	3.15~4.15	≤5.00	≤0.40	≤0.40
Co	C	Ta	Si, Mn	P, S	O	N
≤1.0	≤0.10	≤0.05	each ≤ 0.5	each ≤ 0.05	≤200 ppm	≤200 ppm

**Table 2:** Mechanical properties of Inconel 625 at two strain rates.

Strain Rate	No.	E (GPa)	Yield Stress (MPa)	Tensile Strength (MPa)
5 × 10^−4^ s^−1^	4-1	206.36	1158.35	1649.79
	4-2	202.77	1176.01	1631.73
	Average	204.57	1172.18	1640.76
5 × 10^−5^ s^−1^	5-1	199.76	1107.62	1596.18
	5-2	198.08	1131.28	1569.32
	Average	198.92	1119.45	1582.75

**Table 3:** Hardening index of Inconel 625 at two strain rates.

Strain Rate	No.	Hardening Capacity	*n*_1_	*n*_2_	*n* ^*^
5 × 10^−4^ s^−1^	4-1	0.42	0.14	0.40	0.34
	4-2	0.39	0.15	0.41	0.35
	Average	0.41	0.15	0.41	0.35
5 × 10^−5^ s^−1^	5-1	0.44	0.13	0.35	0.3
	5-2	0.39	0.14	0.41	0.3
	Average	0.41	0.14	0.38	0.3

**Table 4:** Fitting parameters of different model.

Strain Rate	Hybrid Model				Ludwik	H–S
	*c*	*m_2_*	*K*	ER	ER	ER
5 × 10^−4^ s^−1^	1.33	2.9	663	0.48%	0.70%	1.12%
5 × 10^−5^ s^−1^	1.42	2.65	584	0.79%	1.09%	1.01%

**Table 5:** Slip behaviour and CRSS of Inconel 625 at two strain rates.

Slip	Slip	Slip	Schmid	Critical	Critical
System	Plane, n	Direction, s	Factors,	Resolved	Resolved
			|M|	Shear Stress,	Shear Stress,
				*τ_crss_*	*τ_crss_*
				Strain Rates:	Strain Rates:
				5 × 10^−4^ s^−1^	5 × 10^−5^ s^−1^
*γ*_1_	(−1, 1, −1)	[−1, −1, 0]	0.29	337.37	322.19
*γ*_2_		[0, −1, −1]	0.49	573.72	547.91
*γ*_3_		[1, 0, −1]	0.20	236.35	225.72
*γ*_4_	(1, 1, 1)	[−1, 0, 1]	0.27	322.24	307.74
*γ*_5_		[0, −1, 1]	0.42	490.05	468.01
*γ*_6_		[1, −1, 0]	0.14	167.81	160.26
*γ*_7_	(−1, 1, 1)	[0, −1, 1]	0.18	209.38	199.96
*γ*_8_		[1, 0, 1]	0.35	405.90	387.64
*γ*_9_		[1, 1, 0]	0.17	196.53	187.68
*γ*_10_	(1, 1, −1)	[−1, 0, −1]	0.13	152.68	145.82
*γ*_11_		[−1, 1, 0]	0.02	26.97	25.76
*γ*_12_		[0, −1, −1]	0.11	125.71	120.06

**Table 6:** The number of dislocation under both strain rates.

Strain Rate	Grid	Number of Intersections
5 × 10^−4^ s^−1^	1	17.50
	2	21.17
	3	18.17
	4	16.50
	average	18.33
5 × 10^−5^ s^−1^	5	7.33
	6	7.83
	7	6.83
	8	6.50
	average	7.13

## Data Availability

The data presented in this study are available on request from the corresponding author.
